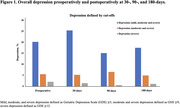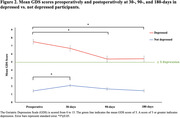# Examining Depression in Older Surgical Patients: An Observational Cohort Study

**DOI:** 10.1002/alz.089860

**Published:** 2025-01-03

**Authors:** Yasmin Alhamdah, Ellene Yan, Nina Butris, Paras Kapoor, Leif Erik Lovblom, David He, Frances Chung

**Affiliations:** ^1^ Toronto Western Hospital, University Health Network, Toronto, ON Canada; ^2^ Temerty Faculty of Medicine, University of Toronto, Toronto, ON Canada; ^3^ Toronto General Hospital, University Health Network, Toronto, ON Canada; ^4^ Mount Sinai Hospital, Toronto, ON Canada

## Abstract

**Background:**

Depression affects individuals across various ages but is of significance in the older surgical population due to its adverse impact on cognitive function, surgical recovery, and overall functional disability. This study aimed to determine the overall prevalence and trajectory of depression in older surgical patients preoperatively, and at 30‐, 90‐ and 180‐days postoperatively.

**Method:**

This study is a prespecified sub‐study and analysis of the Postoperative Functional Disability in Unrecognized Cognitive Impairment Study. Participants ≥ 65 years undergoing elective non‐cardiac surgery were recruited. Participants completed the 15‐item Geriatric Depression Scale (GDS) through an online survey preoperatively and postoperatively at 30‐, 90‐ and 180‐days. A cut‐off of ≥ 5 was used to define depression. Participants also completed four cognitive screening tools: the Telephone Montreal Cognitive Assessment and Modified Telephone Interview for Cognitive Status over the telephone and the Ascertain Dementia Eight‐item Questionnaire and Center for Disease Control and Prevention cognitive question through survey. Cognitive impairment (CI) was defined as meeting the cut‐off on one of the cognitive screening tools. Linear mixed‐effects models were used for trajectory analysis.

**Result:**

Among 307 participants (mean ± SD age: 72.9 ± 5.5; 56.0% female), 62 (20.2%) screened positive for preoperative depression. Forty‐five (14.7%) had mild depression (GDS score 5‐8), 11 (3.6%) had moderate depression (GDS score 9‐11), and 6 (2.0%) had severe depression (GDS score 12‐15). Those who were depressed had significantly lower mean GDS scores at 90‐ and 180‐days postoperatively vs. preoperatively (5.38 ± 0.37 and 5.41 ± 0.35 respectively vs. 7.52 ± 0.28, *P≤*0.05). While the value at 30‐days was lower than that preoperatively, it was not significant. Non‐depressed participants had significantly higher 30‐days postoperative mean GDS score vs. preoperatively (2.09 ± 0.16 vs. 1.42 ± 0.14, *P≤*0.05). The mean GDS scores decreased over time in both groups (*P for time <0.0001*) with significant difference in the trajectories (*P for interaction <0.0001)*. Of those with preoperative depression, 63% screened positive for CI on ≥1 cognitive screening tool vs. 33% without depression.

**Conclusion:**

Depression is prevalent in older surgical patients. Our novel study findings enable better understanding of depression in the older surgical population.